# Cardiac Involvement by HIV-Associated DLBCL

**DOI:** 10.1155/2018/7531319

**Published:** 2018-07-24

**Authors:** Charles T. Mupamombe, Jude Noel, Derek B. Laskar, Liza Valdivia

**Affiliations:** ^1^Department of Internal Medicine, STAR Clinic, SUNY Downstate Medical Center, Brooklyn, NY, USA; ^2^Department of Internal Medicine, SUNY Downstate Medical Center, Brooklyn, NY, USA; ^3^Department of Pathology, SUNY Downstate Medical Center, Brooklyn, NY, USA; ^4^Department of Infectious Diseases, STAR Clinic, SUNY Downstate Medical Center, Brooklyn, NY, USA

## Abstract

Non-Hodgkin's lymphoma (NHL) is a common AIDS-defining malignancy among people living with HIV. Of the different types of NHLs, diffuse large B-cell lymphoma (DLBCL) is the most common. Prognosis of DLBCL has improved over the years in the general population but remains relatively poor in HIV-positive individuals. Almost any organ system can be affected by DLBCL; however, cardiac involvement remains rare and suggests aggressive disease. We present a case of DLBCL in an HIV-positive patient, who had cardiac involvement, with the only clue to cardiac involvement being symptom being tachycardia and dysphagia.

## 1. Introduction

Non-Hodgkin's lymphoma (NHL) is the most frequent neoplastic cause of death in people living with HIV (PLWHIV) and is considered to be AIDS defining [1]. Almost all NHLs in PLWHIV have a casual relation to Epstein–Barr virus infection [2]. Diffuse large B-cell lymphoma (DLBCL) is the most common type of NHL in the general population constituting 40% of all cases globally [2, 3]. Of the AIDS-related lymphomas (ARLs), DLBCL is also the most common, constituting 60–80% of ARLs [4], with a median age found to be 44 years in one study [1]. Subtypes of DLBCL include T-cell/histiocyte-rich B-cell lymphoma, primary DLBCL of the CNS, primary cutaneous DLBCL, EBV-positive DLBCL, and DLBCL not otherwise specified [5]. Further molecular classification based on cell origin defines two molecular subtypes: germinal centre B-cell like (GCB) and activated B-cell like (ABC) [6]. Patients with GCB subtype have better survival than those with ABC [6]. Symptoms of DLBCL are usually nonspecific and can include the typical B-symptoms of fever, night sweats, and weight loss. In general, lymphoma deposits to the heart are relatively rare, with some reporting rates of 13.6% [7]. DLBCL with cardiac involvement is just as rare, if not even more. We present a case of an HIV-positive man, diagnosed with DLBCL at the time of HIV diagnosis with disease recurrence involving the heart.

## 2. Case

**Figure 1:**
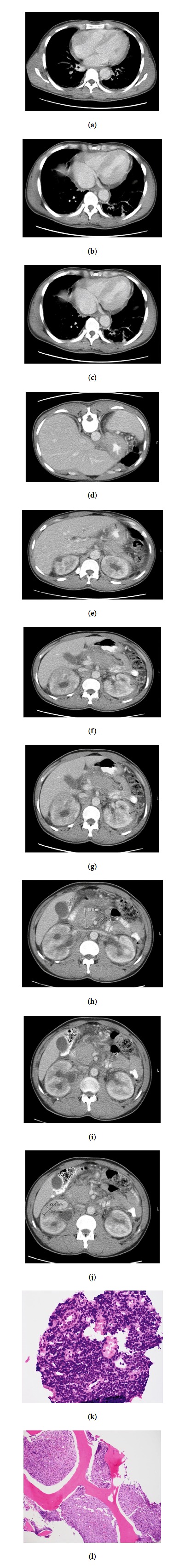
(a–c) CT imaging of the chest without contrast. The heart is normal in size with trace pericardial effusion. Cardiac mass can be appreciated in these images. (d–j) CT abdomen and pelvis with intravenous contrast. The pancreas enlarged without focal mass. (e–j) Multiple soft tissue densities are seen in the left midkidney eroding the renal cortex. There is a mild perirenal soft tissue density rim that can be noted, more prominent in (j), left greater than right. Diffuse mesenteric edema, mild mesenteric lymphadenopathy, is also noted. (k) A high-power slide of kidney mass biopsy showing sheets of large atypical lymphocytes infiltrating the renal interstitium; scattered renal tubules are also seen (H&E, ×400). The atypical cells are immunopositive for CD45, CD20, PAX-5, and CD10, and they are negative for CD3, CD5, CD30, MUM-1, cyclin D1, BCL-2, BCL-6, CD68, and c-myc. The immunoprofile is consistent with diffuse large B-cell lymphoma (DLBCL). (l) Low-power slide of bone marrow biopsy showing bony trabeculae with diffuse infiltration of large atypical lymphocytes and dispersed marrow elements in the background (H&E, ×100). The histomorphology and immunoprofile are similar to infiltrates seen in kidney mass.

A 48-year-old man with no apparent past medical history initially presented with abdominal pain associated with nausea and vomiting. Physical examination was unremarkable. Lab results were significant for anemia, with a hemoglobin level of 11.6 g/dL (14–18), hematocrit 32.2% (42–52), and lipase 164 U/L (13–60). His comprehensive metabolic panel was unrevealing. A CT of the abdomen and pelvis with contrast was performed, which revealed an enlarged pancreas without a focal mass, diffuse surrounding mesenteric edema, mild retroperitoneal lymphadenopathy, mild mesenteric lymphadenopathy, rectal wall thickening with perirectal lymphadenopathy, left renal mass measuring 3.7 × 1.4 × 1.4 cm, right renal mass measuring 2.3 × 4.3 × 5.2 cm, marked diffuse urinary bladder wall thickening, and trace pericardial effusion (Figure 1). The concern at the time was for peritoneal carcinomatosis and/or mesenteric tumor. Urology and oncology services were invited to evaluate the patient, both in agreement for a biopsy of the lymph node; in addition, urine cytology and HIV were tested.

Urine cytology revealed atypical lymphocytes. Renal biopsy revealed diffuse large B-cell lymphoma (DLBCL). Bone marrow biopsy was consistent with involvement of B-cell lymphoma. HTLV-I/II antibody was negative. HIV screening returned with a positive result. Initial CD4 was 440/*μ*l with a percentage of 14%, and HIV RNA viral load by PCR was 61800 copies/mL. Hepatitis C virus screening was negative; hepatitis B screening was positive for the core antibody and surface antibody but negative for surface antigen. His lactate dehydrogenase (LDH) was above the upper limit of the detection for our lab, >2500 U/L (135–225). Cerebrospinal fluid at the time revealed atypical lymphocytes.

Biopsy of the kidney mass revealed atypical lymphocytes positive for CD45, CD20, PAX-5, and CD10, while negative for CD5, CD30, MUM-1, BCL-1 (cyclin D1), BCL-2, BCL-6, c-myc, CD68, CD34, CD117, and MPO, with a high proliferation index (Ki-67) of 80% approximately. The morphology was consistent with diffuse B-cell lymphoma (DLBCL), NOS, and germinal center B-cell subtype involving the kidney. A subsequent bone marrow biopsy revealed involvement by DLBCL (Figure 1).

His Revised International Prognostic Indicator (R-IPI) was calculated to be 4, NCCN-IPI of 5, and CNS-IPI of 4. Decision was made to offer him dose-adjusted rituximab, etoposide, prednisone, vincristine, cyclophosphamide, and doxorubicin (R-EPOCH) to treat his disease. For HIV, he was treated with raltegravir, tenofovir, and emtricitabine, with a subsequent decrease in viral load to our laboratory's threshold for undetectable. This regimen was chosen specifically for the tenofovir to act as prophylaxis to suppress hepatitis B virus reactivation. In addition, he was treated with fluconazole and acyclovir for antimicrobial prophylaxis. He received 3 cycles of chemotherapy and 1 dose of intrathecal methotrexate, with initial improvement in renal lesions. However, after the third cycle, he presented with dysphagia, Bell's palsy, and diffuse bone pain. Neurology service was consulted recommending MRI of the brain without contrast, which was unremarkable. Subsequent CSF flow cytometry was positive for CD10+ B-cells, with a conclusion of DLBCL infiltrating the CNS. Gastroenterology service was consulted, who performed an EGD but found no explanation for dysphagia. ENT service found mucous in the postcricoid region but had no acute interventions to offer, and evaluation by the speech and language pathologist revealed marked pharyngeal dysphagia, with high risk for aspiration.

**Figure 2:**
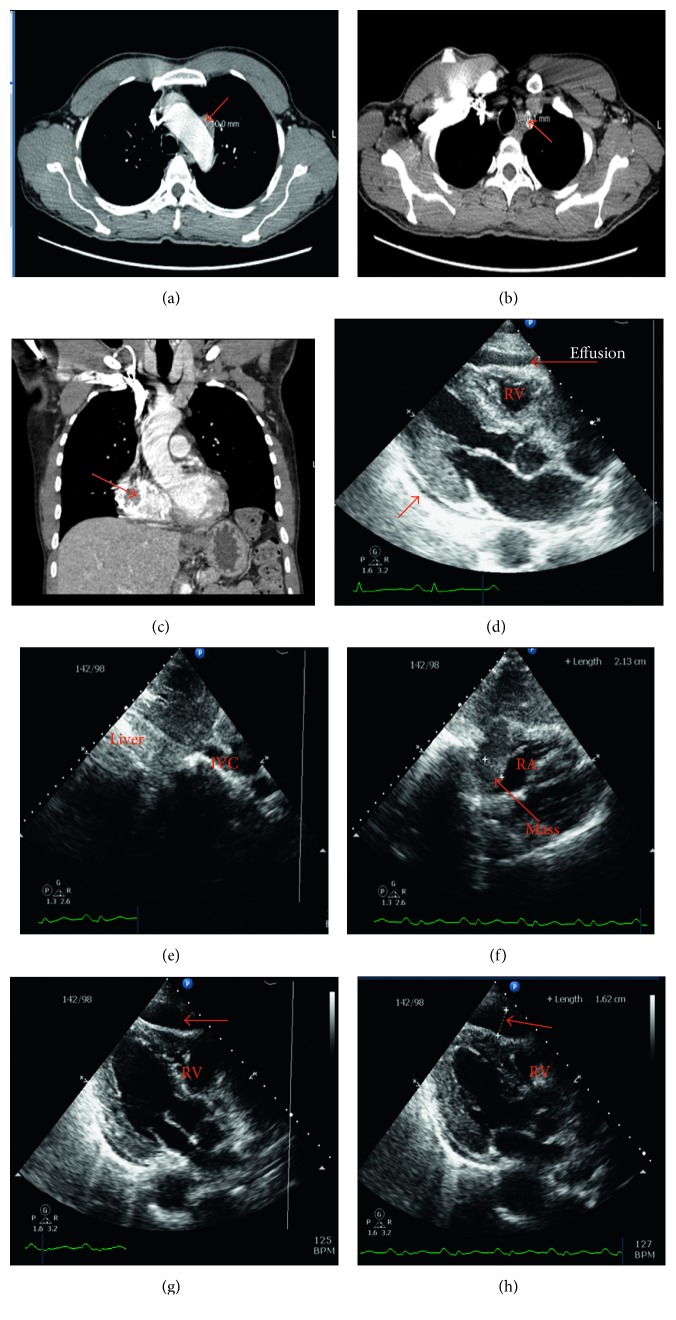
(a–c) CT imaging of the chest without contrast. Illustrates are new paratracheal and pericardiac masses, compared with Figure 1. (d–h) 2D echocardiographic images obtained after results of the CT imaging in (a–c). (d) A small pericardial effusion around the left ventricle, with a larger loculated pericardial effusion compressing the right ventricle. (e) A mass effect on the IVC, which also reduced with inspiration. (f) A mass around the right atrium. (g–h) The loculated pericardial effusion compressing the right ventricle.

A repeat CT of the chest without contrast revealed a right pericardial mass with mediastinal lymphadenopathy. At that time, he did not report any chest pain, dyspnea, or cough. His vital signs were remarkable only for sinus tachycardia of 110 beats per minute, and he was normotensive, tolerated room air, and maintained good mentation. A subsequent echocardiogram revealed a large loculated pericardial effusion near the right ventricle, with echocardiographic evidence of tamponade. A large mass could be appreciated in the right atrium in the lateral wall, possibly obstructing the IVC. Cardiac mass size was 2.1 cm  ×  2.5 cm (Figure 2). This was concerning for disease recurrence. He was transferred to the coronary care unit for monitoring but did not show any clinical signs of cardiac tamponade. Treatment options were discussed with the patient, who opted for palliative care measures and a transfer to hospice care.

The patient's wishes were followed. A PEG tube was placed for nourishment, and his pain was controlled with opioid analgesics. Plans to transfer him to inpatient hospice care were put in place. Unfortunately, the patient expired before he could be discharged, 5 months following initial diagnosis of DLBCL, and within 3 weeks of pericardial involvement being discovered. His dysphagia never resolved and was likely related to the discovered cardiac mass.

## 3. Discussion

Cardiac involvement by lymphoma at autopsy has been described in 16% of patients with Hodgkin's disease, 18% with NHL, and occurring at a median of 20 months after initial diagnosis [7]. Symptoms of involvement include heart failure, pericardial pain, rhythm abnormalities, cardiac tamponade, and even superior vena cava syndrome [8]. O'Mahony et al. described a case of DLBCL involving the heart, in a patient with multiple recurrences and succumbed to disease within a year of diagnosis [7]. Vivekanandarajah et al. described a case of DLBCL diagnosed as a presentation of HIV, with good response to CHOP chemotherapy, and the patient was reported alive at the time of their publication [9]. Another case in Danish reported of an HIV-positive Somali woman with cardiac involvement by DLBCL [10]. The cases mentioned, including our own, suggest a very poor response to treatment when there is cardiac involvement, and in turn a poor prognosis, as only one seems to have been alive at the time of report.

In general, DLBCL has a slight male predominance [1, 11], with a 5-year survival estimated at 62% with no bone marrow involvement, but only 10% when the bone marrow is involved [12]. The 5-year survival differs with age: 0–19 years reported 86.4%; 20–64 years reported 89.4%; and 65+ years reported 50.7% [11]. Genes that have been associated with DLBCL include 3q27 as a susceptibility locus, activation of NF-*κ*B pathway in the activated B-cell-like type, and recurrent mutations targeting histone-modifying genes [2]. Since rituximab has been added to standard therapy, BCL-2 and BCL-6 genes are no longer considered important prognostic genetic factors [13]. In persons living with HIV (PLWHIV), the degree of immunosuppression is the most important risk factor for NHL onset, but other independent risk factors include uncontrolled HIV viral load and age over 50 [14]. PLWHIV often have more aggressive DLBCL and substantially lower survival compared to their HIV-negative counterparts [14].

Prognostic tools are used to help identify aggressive disease and predict outcomes to therapy. The Revised International Prognostic Indicator (R-IPI) classifies patients into 3 risk groups, with incremental 4-year progression-free survival (PFS) the less risk factors that are identified [13]. With the R-IPI, a score of 0 is associated with 94% 4-year PFS, a score of 1-2 is associated with 80% 4-year PFS, and a score of 3–5 is associated with a 53% 4-year PFS. The NCCN-IPI was created using a larger cohort and is a more powerful tool than the R-IPI at predicting survival and discriminating high- and low-risk patients [15]. In addition, a CNS-IPI score is used to predict high risk for CNS relapse and help us to determine directed investigations and prophylactic interventions. The high-risk group CNS-IPI has been shown to have a >10% risk of CNS relapse [16].  Table 1 shows a comparison of prognostic tools. Although not initially designed for PLWHIV, the R-IPI has been shown to still be useful in this population and can still be applied [17]. For NHL in general, PFS and overall survival for PLWHIV have improved markedly in the modern combined antiretroviral therapy (cART) era compared to pre-cART, and we now know that CD4 cell count is no longer a major determinant in survival for HIV-associated lymphoma [14] but may still contribute to poor outcomes with severe immunosuppression defined as CD4 <200 increasing the hazard of death [18]. Interestingly, Besson et al. did not find significant differences in outcomes between PLWHIV diagnosed with NHL receiving R-CHOP and those who were HIV negative, despite a higher rate of aggressive presentation for PLWHIV [14].

In their study, Riedel et al. found DLBCL to be the most common lymphoma in the cART era, with a reported rate of 41% among those studied [18]. Overall survival at 1 year for DLBCL was found to be lower than Burkitt's lymphoma but higher than Hodgkin's and other lymphomas in this study. Their findings were similar to other reports in the literature [17, 20]. Even with significant improvements in chemotherapy and supportive care, PLWHIV were found to have poor survival, mainly predicted by poor performance status, severe immune compromise, and advance stage disease at presentation [18]. Interestingly, HIV-related features such as CD4 count were found to have no bearing in achieving complete response to chemotherapy in some patients [1]. Studies in the literature are in agreement that PLWHIV seem to present with a disproportionately higher percentage of stage III/IV disease compared to their HIV-negative counterparts, and even though outcomes are improving, they are still worse compared to that of HIV-negative patients. Even though some NHLs in PLWHIV can be associated with EBV, EBV positivity has no impact on survival.

Shen et al. suggested CHOP to not be enough for HIV-associated DLBCL, opting for escalated regimens such as DA-EPOCH or CHOP-E [17]. Baptista et al. found that combining chemotherapy with cART achieved similar complete response and disease-free survival rates in the HIV cohort compared to their negative counterparts with DLBCL [1]. Coutinho et al. concluded that HIV status has no bearing on chemotherapy for DLBCL in PLWHIV [4]. With a strong base of evidence, these patients can be treated very similarly to their HIV-negative counterparts, with improved outcomes compared pre-cART approaching that of the general population. The NCCN recommend dose-adjusted R-EPOCH as the preferred therapy, with rituximab not indicated if CD20 negative and advice to consider eliminating rituximab if CD4 <100. To improve outcomes, cART should be started or continued. Comorbid conditions, co-occuring infections such as hepatitis B virus, and drug interactions should be considered, and cART tailored for all these factors.

## 4. Conclusion

DLBCL remains a common AIDS-defining malignancy. Cardiac involvement suggests a particularly aggressive disease with a poor prognosis. Treatment approach for HIV-positive patients with DLBCL should incorporate controlling the virus in addition to chemotherapy for DLBCL. Prognostic tools used for HIV-negative patients with DLBCL such as the IPI are applicable to HIV-positive patients; however, more work is needed to identify why DLBCL remains with a poorer prognosis in PLWHIV compared to their HIV-negative counterparts.

## Figures and Tables

**Table 1:** Factors included in calculating the R-IPI, NCCN-IPI, and CNS-IPI [16, 19].

R-IPI	NCCN-IPI	CNS-IPI
Age >60 years Elevated LDH Two or more extranodal sites of disease Ann Arbor stage 3 or more ECOG score 3 or more	Patient age in years (points): (i) Up to 40 (ii) 41 to 60 (1) (iii) 61 to 75 (2) (iv) >75 (3)	Kidney and/or adrenal glands involved Age >60 LDH above normal ECOG >1 State III/IV disease Extranodal involvement
	Ann Arbor stage: (i) I or II (ii) III or IV (1)	
	ECOG/WHO performance status (i) 0 or 1 (ii) 2–4 (1)	
	Patient serum LDH (U/L) ratio: (i) 1–3 (1) (ii) >3 (2)	
	Extranodal disease (bone marrow, CNS, liver, GI tract, lung) (1)	
	Total score of up to 8	
